# Statistical Inference for Alpha-Series Process with the Generalized Rayleigh Distribution

**DOI:** 10.3390/e21050451

**Published:** 2019-04-30

**Authors:** Hayrinisa Demirci Biçer

**Affiliations:** Arts and Sciences Faculty, Statistics Department, University of Kirikkale, Kirikkale 71450, Turkey; hdbicer@kku.edu.tr

**Keywords:** alpha-series process, geometric process, maximum likelihood estimate, modified maximum spacing estimate, modified least-squares estimate

## Abstract

In the modeling of successive arrival times with a monotone trend, the alpha-series process provides quite successful results. Both selecting the distribution of the first arrival time and making an optimal statistical inference play a crucial role in the modeling performance of the alpha-series process. In this study, when the distribution of the first arrival time is the generalized Rayleigh, the problem of statistical inference for the α, β, and λ parameters of the alpha-series process is considered. Further, in order to obtain optimal modeling performance from the mentioned alpha-series process, various estimators for the model parameters are obtained by employing different estimation methodologies such as maximum likelihood, modified maximum spacing, modified least-squares, modified moments, and modified L-moments. By a series of Monte Carlo simulations, the estimation efficiencies of the obtained estimators are evaluated through the different sample sizes. Finally, two real datasets are analyzed to illustrate the importance of modeling with the alpha-series process.

## 1. Introduction

Currently, modeling the failure times of an engineering product or a system is quite important in terms of reliability. It is a general approach to use the renewal process in modeling the non-trending times of successive failures (successive arrival times) of repairable systems. However, in most cases, successive arrival times for repairable systems may include a trend due to the effects of accumulated wear, aging, or unknown reasons such as changing the maintenance unit and quality of replacement parts. In this case, it would be more appropriate to consider a model with monotonic behavior, which takes into account the trend in the data [1].

Lam [2] introduced the geometric process (GP) for modeling the successive arrival times with a monotone trend. In the literature, there is a wide range of studies to show the main characteristics of the GP and its performance in the modeling of successive arrival times with a trend; see [1,3,4,5,6,7,8,9,10,11,12,13,14,15,16,17]. If the GP cannot model the data, the alpha-series process (ASP) can be employed for modeling the successive arrival times. The ASP was introduced as a strong alternative to the GP by Braun et al. [18] and is described by the following definition.

**Definition** **1.** *Let $X_{k}$ be the interarrival time of the ${\left(k-1\right)}^{th}$ and $k^{th}$ events of a counting process $\left\{N(t),t\geq 0\right\}$ for k=1,2,…. The process $\left\{X_{k},k=1,\ldots ,n\right\}$ is said to be an ASP with parameter α if there exists a real number such that the random variables:*

(1) $$Y_{k}=k^{\alpha }X_{k},k=1,2,\ldots$$

*are independently and identically distributed (iid) with the distribution function F; see [19].*

**Figure 1:**
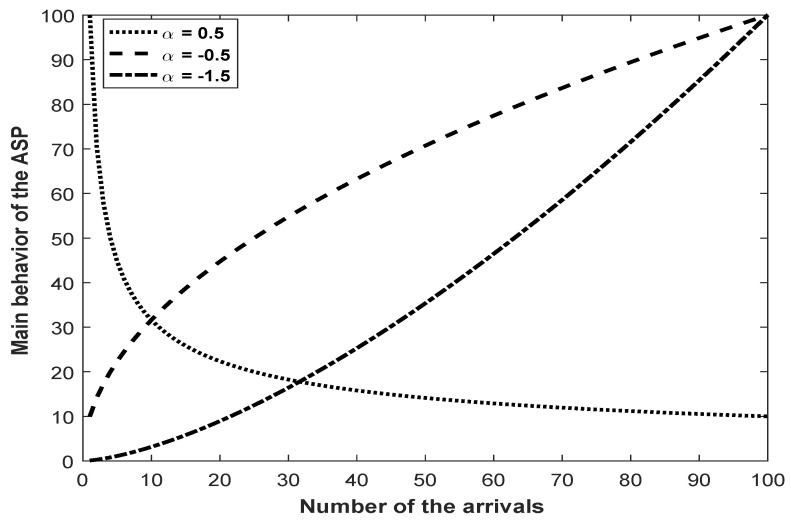
Behavior of the alpha-series process (ASP).

The parameter α manages the monotonic behavior of the ASP. For the different values of the parameter α, the behavior of the ASP is illustrated in Figure 1.

As can be clearly seen from Figure 1, the behavior of the ASP is monotonically decreasing when α>0, monotonically increasing with decreasing slope when −1<α<0, and monotonically increasing with increasing slope when α<−1. If α=0, then the ASP is equivalent to the renewal process (RP). In this context, ASP is a more capable model than the GP for more various data types; because the GP does not have the capability of modeling data that are monotonically increasing with decreasing slope. Furthermore, some useful properties and theoretical results for the ASP can be found in [18,20].

An ASP contains two types of parameters, which are process parameter α and the distributional parameters of the first arrival time $X_{1}$. Estimation of these parameters is quite important since they determine the mean and variance of random variable $X_{k},\left(k=1,2,\cdots ,\right)$ in such a way that:

(2) $$E\left(X_{k}\right)=\frac{\mu }{k^{\alpha }}\;\;\;\;\;\;\;\;k=1,2,\ldots$$

(3) $$Var\left(X_{k}\right)=\frac{\sigma^{2}}{k^{2\alpha }},k=1,2,\ldots ,$$

where μ and $\sigma^{2}$ are the expectation and variance of the first arrival time $X_{1}$, respectively. Although the ASP given by Definition 1 has many useful properties and superior data modeling capability, it has not been able to achieve its deserved position in the literature due to the popularity of the GP in reliability and scheduling problems. Nevertheless, one can find several papers on statistical inference for the ASP; see [19,21,22].

The main goal of the present study is to examine the solution of the parameter estimation problem for ASP under assumption that the first arrival time $X_{1}$ is distributed generalized Rayleigh, which is a possible alternative to popular reliability distributions such as Weibull, gamma, and log normal. The generalized Rayleigh distribution has special importance among of lifetime distributions since its hazard rate function can be either a bathtub type or an increasing function, depending on the shape parameter. The hazard function of the distribution is a bathtub type when its shape parameter is less than or equal to 1/2 and is an increasing function when its shape parameter is greater than 1/2 [23]. Therefore, it can be applied to many life testing experiments in which the aging effect is expected [3].

The rest of this paper is organized as follows: in Section 2, estimators for the parameters of ASP with the generalized Rayleigh distribution are obtained using the different estimation methodologies such as maximum likelihood (ML), modified maximum spacing (MMSP), modified least-squares (MLS), modified moments (MM), and modified L-moments (MLM). Some Monte Carlo simulation results, which compare the estimation efficiencies of the estimators obtained in Section 2, are presented in Section 3. Section 4 includes two real data applications that show the superiority of the ASP with the generalized Rayleigh distribution according to both the RP and the various ASPs with the gamma, Weibull, log normal, and inverse Gaussian. Section 5 concludes the study.

## 2. Estimation of the Parameters of ASP with the Generalized Rayleigh Distribution

In this section, we consider the parameter estimation problem for ASP with the generalized Rayleigh distribution by using the different estimation procedures such as the ML, MMSP, MLS, MM, and MLM.

Before progressing to the estimation stage, let us recall the generalized Rayleigh distribution, also known as two-parameter Burr Type X distribution [23]. The probability density function of the generalized Rayleigh distribution is:

(4) $$f\left(x,\beta ,\lambda \right)=2\beta \lambda^{2}xe^{-{\left(\lambda x\right)}^{2}}{\left(1-e^{-{\left(\lambda x\right)}^{2}}\right)}^{\beta -1},\;\;\;\;\;x>0,$$

and the corresponding cumulative distribution function (cdf) is:

(5) $$F\left(x,\beta ,\lambda \right)={\left(1-e^{-{\left(\lambda x\right)}^{2}}\right)}^{\beta }\;\;\;\;\;x>0,$$

where β>0 and λ>0 are the shape and scale parameters of the distribution, respectively. From now on, the generalized Rayleigh distribution with parameters β and λ will be indicated as $GR\left(\beta ,\lambda \right)$.

Shannon entropy is a very important inferential measure to explain the variability or uncertainty of a random variable. The Shannon entropy for a random variable *X* with pdf *f* is given by (see [24,25]),

(6) $$H\left(X\right)=E\left\{-lnf(x)\right\}.$$

**Figure 2:**
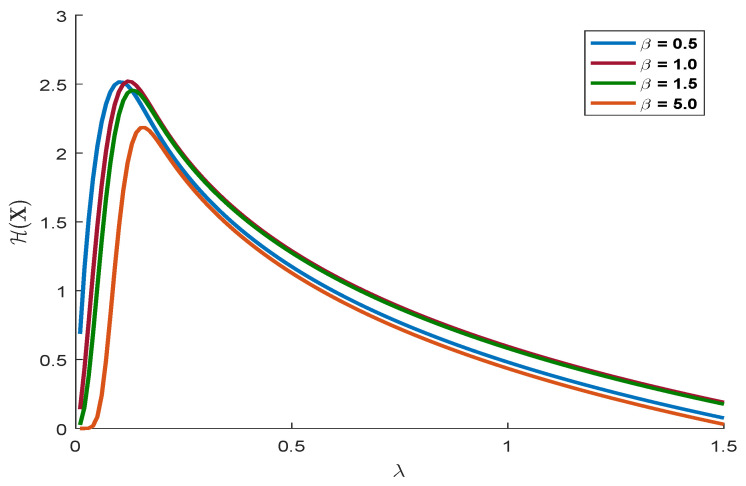
Shannon entropy of the generalized Rayleigh distribution for different values of the parameters.

Using the pdf (4) in the equation (6), the Shannon entropy of the generalized Rayleigh distribution is found as:

(7) $$\begin{array}{cc} H\left(X\right) & =-\int_{0}^{\infty }\ln\left(2\beta \lambda^{2}xe^{-{\left(\lambda x\right)}^{2}}{\left(1-e^{-{\left(\lambda x\right)}^{2}}\right)}^{\beta -1}\right)f\left(x\right)dx\;\\ & =-\left(\ln2+\ln\beta +2\ln\lambda -\lambda^{2}\int_{0}^{\infty }x^{2}f\left(x\right)dx+\int_{0}^{\infty }\left(\ln x\right)f\left(x\right)dx+\left(\beta -1\right)\int_{0}^{\infty }\ln\left(1-e^{-{\left(\lambda x\right)}^{2}}\right)f\left(x\right)dx\right)\;\\ & =-\left(\ln2+\ln\beta +2\ln\lambda -\lambda^{2}\frac{\left(\Psi \left(\beta +1\right)-\Psi \left(1\right)\right)}{\lambda^{2}}+\int_{0}^{\infty }\left(\ln x\right)f\left(x\right)dx+\left(\beta -1\right)\int_{0}^{\infty }\ln\left(1-e^{-{\left(\lambda x\right)}^{2}}\right)f\left(x\right)dx\right)\;\\ & =\left(\Psi \left(\beta +1\right)-\Psi \left(1\right)\right)+\left(\frac{\beta -1}{\beta }\right)-\ln2-\ln\beta -2\ln\lambda -\kappa , \end{array}$$

where $f\left(x\right)$ is the pdf of the generalized Rayleigh distribution, $\kappa =E\left(lnX\right)$, and $\Psi \left(.\right)$ is the digamma function [26]. For illustrative purposes, we present Figure 2 where the plots of the Shannon entropy of the generalized Rayleigh distribution are displayed for different values of the parameters.

See also [23,27,28,29] in the context of theoretical properties and estimation problems for the generalized Rayleigh distribution.

### 2.1. Maximum Likelihood Estimation

Let $X_{1},X_{2},\ldots ,X_{n}$ be a random sample from the ASP with parameter α and $X_{1}∼GR\left(\beta ,\lambda \right)$ with the pdf (4). The log-likelihood function lnL(α,β,λ) of the random variables $X_{k},$$\left(k=1,2,\ldots ,n\right)$ can be written as:

(8) $$\begin{array}{cc} \ln L(\alpha ,\beta ,\lambda )= & n\left(\ln2+\ln\beta +2\ln\lambda \right)+2\alpha \ln\left(\Gamma \left(n+1\right)\right)+\;\\ & \sum_{k=1}^{n}\ln x_{k}-\lambda^{2}\sum_{k=1}^{n}{\left(k^{\alpha }x_{k}\right)}^{2}+\left(\beta -1\right)\sum_{k=1}^{n}\ln\left(1-e^{-{\left(\lambda k^{\alpha }x_{k}\right)}^{2}}\right). \end{array}$$

By deriving the (8) log-likelihood function with respect to parameters α,β, and λ, the three normal equations become:

(9) $$\begin{array}{cc} \frac{\partial \ln L(\alpha ,\beta ,\lambda )}{\partial \alpha }= & 2\ln\Gamma \left(n+1\right)-2\lambda^{2}\sum_{k=1}^{n}{\left(k^{\alpha }x_{k}\right)}^{2}\left(\ln k\right)+\;\\ & 2\lambda^{2}\left(\beta -1\right)\sum_{k=1}^{n}\frac{{\left(k^{\alpha }x_{k}\right)}^{2}e^{-{\left(\lambda k^{\alpha }x_{k}\right)}^{2}}\left(\ln k\right)}{1-e^{-{\left(\lambda k^{\alpha }x_{k}\right)}^{2}}}=0\; \end{array}$$

(10) $$\begin{array}{cc} \frac{\partial \ln L(\alpha ,\beta ,\lambda )}{\partial \lambda }= & \frac{2n}{\lambda }+2\lambda \left(\beta -1\right)\sum_{k=1}^{n}\frac{{\left(k^{\alpha }x_{k}\right)}^{2}e^{-{\left(\lambda k^{\alpha }x_{k}\right)}^{2}}}{1-e^{-{\left(\lambda k^{\alpha }x_{k}\right)}^{2}}}-2\lambda \sum_{k=1}^{n}{\left(k^{\alpha }x_{k}\right)}^{2}=0\; \end{array}$$

(11) $$\begin{array}{cc} \frac{\partial \ln L(\alpha ,\beta ,\lambda )}{\partial \beta }= & \frac{n}{\beta }+\sum_{k=1}^{n}\ln\left(1-e^{-{\left(\lambda k^{\alpha }x_{k}\right)}^{2}}\right)=0.\; \end{array}$$

Unfortunately, these normal equations cannot be solved with respect to the corresponding parameters, and explicit expressions of the ML estimators cannot be obtained from these normal equations. However, Equations (9)–(11) can be solved simultaneously by using a numerical method. Newton’s method is a commonly-used numerical method to investigate the solution of likelihood functions that cannot be solved analytically. Now, let us investigate the ML estimates of the parameters α,β, and λ employing Newton’s method.

Newton’s iterative formula is given by:

(12) $${\hat{\theta }}_{j+1}={\hat{\theta }}_{j}-H^{-1}\left({\hat{\theta }}_{j}\right)\nabla \left({\hat{\theta }}_{j}\right),$$

where *j* is the iteration number, ${\hat{\theta }}_{j}$ is the estimation of the parameter vector in the $j^{\mathrm{th}}$ iteration, $\nabla \left({\hat{\theta }}_{j}\right)$ is the corresponding gradient, and $H\left({\hat{\theta }}_{j}\right)$ is the corresponding Hessian matrix. For our problem, ${\hat{\theta }}_{j},$ $\nabla \left({\hat{\theta }}_{j}\right)$, and $H\left({\hat{\theta }}_{j}\right)$ are defined as:

$${\hat{\theta }}_{j}=\left[\begin{array}{c} {\hat{a}}_{j}\\ {\hat{\lambda }}_{j}\\ {\hat{\beta }}_{j} \end{array}\right],$$

$$\nabla \left({\hat{\theta }}_{j}\right)={\left[\begin{array}{c} \frac{\partial \ln L(a,\lambda ,\beta )}{\partial a}\\ \frac{\partial \ln L(a,\lambda ,\beta )}{\partial \lambda }\\ \frac{\partial \ln L(a,\lambda ,\beta )}{\partial \beta } \end{array}\right]}_{a={\hat{a}}_{j},\lambda ={\hat{\lambda }}_{j},\beta ={\hat{\beta }}_{j}}$$

and:

$$H\left({\hat{\theta }}_{j}\right)={\left[\begin{array}{ccc} \frac{\partial^{2}\ln L\left(a,\lambda ,\beta \right)}{\partial a^{2}} & \frac{\partial^{2}\ln L\left(a,\lambda ,\beta \right)}{\partial a\partial \lambda } & \frac{\partial^{2}\ln L\left(a,\lambda ,\beta \right)}{\partial a\partial \beta }\\ \frac{\partial^{2}\ln L\left(a,\lambda ,\beta \right)}{\partial a\partial \lambda } & \frac{\partial^{2}\ln L\left(a,\lambda ,\beta \right)}{\partial \lambda^{2}} & \frac{\partial^{2}\ln L\left(a,\lambda ,\beta \right)}{\partial \lambda \partial \beta }\\ \frac{\partial^{2}\ln L\left(a,\lambda ,\beta \right)}{\partial a\partial \beta } & \frac{\partial^{2}\ln L\left(a,\lambda ,\beta \right)}{\partial \lambda \partial \beta } & \frac{\partial^{2}\ln L\left(a,\lambda ,\beta \right)}{\partial \beta^{2}} \end{array}\right]}_{a={\hat{a}}_{j},\lambda ={\hat{\lambda }}_{j},\beta ={\hat{\beta }}_{j}},$$

respectively. The elements of the gradient vector $\nabla \left(\theta \right)$ are given in Equations (9)–(11). The elements of the matrix $H\left(\theta \right)$, say $h_{ij}$ $\left(i,j=1,2,3\right)$, are obtained as:

$$\begin{array}{cc} h_{11}=\frac{\partial^{2}\ln L}{\partial \alpha^{2}}= & -4\lambda^{2}\sum_{k=1}^{n}{\left(k^{\alpha }x_{k}\right)}^{2}{\left(\ln k\right)}^{2}-\;\\ & 4\lambda^{2}\left(\beta -1\right)\sum_{k=1}^{n}\frac{{\left(k^{\alpha }x_{k}\right)}^{2}e^{-{\left(\lambda k^{\alpha }x_{k}\right)}^{2}}{\left(\ln k\right)}^{2}}{{\left(e^{-{\left(\lambda k^{\alpha }x_{k}\right)}^{2}}-1\right)}^{2}}\left(e^{-{\left(\lambda k^{\alpha }x_{k}\right)}^{2}}+{\left(\lambda k^{\alpha }x_{k}\right)}^{2}-1\right) \end{array}$$

$$\begin{array}{cc} h_{12}=h_{21}=\frac{\partial^{2}\ln L}{\partial \alpha \partial \lambda }= & -4\lambda \sum_{k=1}^{n}{\left(k^{\alpha }x_{k}\right)}^{2}\left(\ln k\right)-\;\\ & 4\lambda \left(\beta -1\right)\left(\sum_{k=1}^{n}\frac{\lambda^{2}{\left(k^{\alpha }x_{k}\right)}^{4}e^{-{\left(\lambda k^{\alpha }x_{k}\right)}^{2}}\left(\ln k\right)}{{\left(e^{-{\left(\lambda k^{\alpha }x_{k}\right)}^{2}}-1\right)}^{2}}+\sum_{k=1}^{n}\frac{{\left(k^{\alpha }x_{k}\right)}^{2}e^{-k^{2\alpha }\lambda^{2}x_{k}^{2}}\left(\ln k\right)}{e^{-{\left(\lambda k^{\alpha }x_{k}\right)}^{2}}-1}\right) \end{array}$$

$$\begin{array}{cc} h_{13}=h_{31}=\frac{\partial^{2}\ln L}{\partial \alpha \partial \beta }= & -2\lambda^{2}\sum_{k=1}^{n}\frac{{\left(k^{\alpha }x_{k}\right)}^{2}e^{-{\left(\lambda k^{\alpha }x_{k}\right)}^{2}}\left(\ln k\right)}{e^{-{\left(\lambda k^{\alpha }x_{k}\right)}^{2}}-1}\; \end{array}$$

$$\begin{array}{cc} h_{22}=\frac{\partial^{2}\ln L}{\partial \lambda^{2}}= & -\frac{2n}{\lambda^{2}}-4\lambda^{2}\left(\beta -1\right)\sum_{k=1}^{n}\frac{{\left(k^{\alpha }x_{k}\right)}^{4}e^{-{\left(\lambda k^{\alpha }x_{k}\right)}^{2}}}{{\left(e^{-{\left(\lambda k^{\alpha }x_{k}\right)}^{2}}-1\right)}^{2}}-\;\\ & 2\left(\beta -1\right)\sum_{k=1}^{n}\frac{{\left(k^{\alpha }x_{k}\right)}^{4}e^{-{\left(\lambda k^{\alpha }x_{k}\right)}^{2}}}{{\left(e^{-{\left(\lambda k^{\alpha }x_{k}\right)}^{2}}-1\right)}^{2}}-2\sum_{k=1}^{n}{\left(k^{\alpha }x_{k}\right)}^{2} \end{array}$$

$$\begin{array}{cc} h_{23}=h_{32}=\frac{\partial^{2}\ln L}{\partial \lambda \partial \beta }= & -2\lambda \sum_{k=1}^{n}\frac{{\left(k^{\alpha }x_{k}\right)}^{2}e^{-{\left(\lambda k^{\alpha }x_{k}\right)}^{2}}}{e^{-{\left(\lambda k^{\alpha }x_{k}\right)}^{2}}-1}\; \end{array}$$

$$\begin{array}{cc} h_{33}=\frac{\partial^{2}\ln L}{\partial \beta^{2}}= & -\frac{n}{\beta^{2}}\; \end{array}$$

Note that *H* is a symmetrical matrix. We can also compute the inverse of the matrix *H* by:

$$H^{-1}=\frac{1}{Det\left(H\right)}\left[\begin{array}{ccc} h_{22}h_{33}-h_{23}h_{32} & -h_{12}h_{33}-h_{13}h_{32} & h_{12}h_{23}-h_{13}h_{22}\\ -h_{21}h_{33}-h_{31}h_{23} & h_{11}h_{33}-h_{13}h_{31} & -h_{11}h_{23}-h_{21}h_{13}\\ h_{21}h_{32}-h_{22}h_{31} & -h_{11}h_{32}-h_{12}h_{31} & h_{11}h_{22}-h_{12}h_{21} \end{array}\right],$$

where $Det\left(H\right)=h_{11}h_{22}h_{33}-h_{11}h_{23}h_{32}-h_{12}h_{21}h_{33}+h_{12}h_{31}h_{23}+h_{21}h_{13}h_{32}-h_{13}h_{22}h_{31}$. Hence, we can estimate the parameter vector θ using the iterative method given by Equation (12) with an initial estimation ${\hat{\theta }}_{0}$. Then, the ML estimates of the parameters α, λ and β, say ${\hat{\alpha }}_{ML},$${\hat{\lambda }}_{ML}$ and ${\hat{\beta }}_{ML},$ respectively, are obtained as respective elements of the ${\hat{\theta }}_{m+1}$.

### 2.2. Modified Methods

In the problem of estimating the parameters of an ASP, an explicit expression of the parametric estimator of the parameter α may not always be obtained, as in our case. In such a case, the parameter α is parametrically estimated using numerical methods. However, some divergence problems may be encountered in solving the parametric estimator by using numerical methods. In order to avoid these divergence problems in the parametric estimation of the parameter α and to provide an appropriate initial value for numerical methods, the parameter α can be estimated non-parametrically by using equation,

(13) $${\hat{\alpha }}_{NL}=\frac{\sum_{k=1}^{n}\ln k\sum_{k=1}^{n}\ln X_{k}-n\sum_{k=1}^{n}\ln X_{k}\ln k}{n\sum_{k=1}^{n}{\left(\ln k\right)}^{2}-{\left(\sum_{k=1}^{n}\ln k\right)}^{2}}.$$

For further information on deriving the estimator ${\hat{\alpha }}_{NP}$, we refer the readers to [21].

Furthermore, when the ${\hat{\alpha }}_{NP}$ given by Equation (13) yields Equation (1), we have:

(14) $${\hat{Y}}_{k}=k^{{\hat{\alpha }}_{NL}}X_{k}.$$

Thus, using Equation (14) and the estimator ${\hat{\alpha }}_{NP}$, the other distributional parameters of the ASP can be estimated by using a selected method such as maximum spacing, least-squares, moments, or L-moments. This estimation rule is known as the modified estimation rule in the literature.

#### 2.2.1. MMSP Estimation

In this subsection, we use a method based on maximizing the spacings to estimate the unknown parameters λ and β, when the parameter α is estimated by the estimator (13). This method is known as the maximum spacing (MSP) or maximum product space estimation. The MSP estimators have nice properties such as consistency and asymptotic unbiasedness. We refer the readers to [30] and [31] for further information on MSP.

Let $X_{1},X_{2},\cdots ,X_{n}$ be a random sample from the ASP with the parameter α and $X_{1}∼GR\left(\beta ,\lambda \right)$. Furthermore, let the parameter α be estimated as $\alpha_{NL}$ by using the estimator (13). Thus, we have the estimated observations ${\hat{Y}}_{1}$, ${\hat{Y}}_{2}$, …, ${\hat{Y}}_{n}$ from Equation (14). Then, using the same notation as in the paper [31], the MMSP estimators of the parameters β and λ are obtained maximizing:

(15) $$\begin{array}{c} \sum_{j=1}^{n+1}\ln\left[F\left({\hat{Y}}_{\left(j\right)},\lambda ,\beta \right)-F\left({\hat{Y}}_{\left(j-1\right)},\lambda ,\beta \right)\right]\;\\ =\sum_{j=1}^{n+1}\ln\left[{\left(1-e^{-{\left(\lambda {\hat{y}}_{\left(j\right)}\right)}^{2}}\right)}^{\beta }-{\left(1-e^{-{\left(\lambda {\hat{y}}_{\left(j-1\right)}\right)}^{2}}\right)}^{\beta }\right] \end{array}$$

with respect to parameters λ and β, where $F\left(.,\lambda ,\beta \right)$ represent the cdf of the generalized Rayleigh distribution given by Equation (5), and ${\hat{Y}}_{\left(j\right)}$s $\left(j=1,2,\ldots ,n\right)$ represent the ordered ${\hat{Y}}_{j}$s, $\left(j=1,2,\ldots ,n\right)$. ${\hat{Y}}_{\left(0\right)}=0$ and ${\hat{Y}}_{\left(n+1\right)}=\infty$.

#### 2.2.2. MM Estimation

Let $X_{1},X_{2},\cdots ,X_{n}$ be a random sample from the ASP with parameter α and $X_{1}∼GR\left(\beta ,\lambda \right)$. In addition, we assume that the parameter α is nonparametrically estimated by (13). By these assumptions, we have the sample ${\hat{Y}}_{1}$, ${\hat{Y}}_{2}$, …, ${\hat{Y}}_{n}$ estimated with Equation (14).

In a general point of view, the moment estimators are obtained equating the first and second population moments to the corresponding sample moments for a family of distributions with two unknown parameters. Unfortunately, the first population moment of the generalized Rayleigh distribution cannot be obtained analytically. Kundu and Ragab [23] obtained the moments estimators for the parameters λ and β by equating the second and fourth population moments of the generalized Rayleigh distribution to the corresponding sample moments. Now, we adapt their approximation to our problem. For the sample ${\hat{Y}}_{1}$, ${\hat{Y}}_{2}$, …, ${\hat{Y}}_{n}$, the second and fourth sample moments, $m_{2}$ and $m_{4},$ are calculated by:

(16) $$m_{2}=\frac{1}{n}\sum_{k=1}^{n}k^{2{\hat{\alpha }}_{NP}}x_{k}^{2}=\frac{1}{n}\sum_{k=1}^{n}{\hat{y}}_{k}^{2}$$

and:

(17) $$m_{4}=\frac{1}{n}\sum_{k=1}^{n}k^{4{\hat{\alpha }}_{NP}}x_{k}^{4}=\frac{1}{n}\sum_{k=1}^{n}{\hat{y}}_{k}^{4},$$

respectively. On the other hand, the second and fourth population moments of the distribution GR(β,λ), say $\mu_{2}$ and $\mu_{4}$, can be easily written as:

(18) $$\mu_{2}=\frac{1}{\lambda^{2}}\left(\Psi \left(\beta +1\right)-\Psi \left(1\right)\right)$$

and:

(19) $$\mu_{4}=\frac{1}{\lambda^{4}}\left(\Psi^{\prime }\left(1\right)-\Psi^{\prime }\left(\beta +1\right)\right)+{\left(\frac{1}{\lambda^{2}}\left(\Psi \left(\beta +1\right)-\Psi \left(1\right)\right)\right)}^{2},$$

respectively, where $\Psi \left(.\right)$ and $\Psi^{\prime }\left(.\right)$ indicate the digamma and polygamma functions, respectively. Then, the MM estimator of the parameter β, ${\hat{\beta }}_{MM}$, can be obtained from the solution of the following nonlinear equation:

(20) $$\frac{\Psi^{\prime }\left(1\right)-\Psi^{\prime }\left(\beta +1\right)}{{\left(\Psi \left(\beta +1\right)-\Psi \left(1\right)\right)}^{2}}-\frac{V}{m_{2}^{2}}=0$$

where $V=m_{4}-m_{2}^{2}$. Furthermore, the MM estimator of the parameter λ, say ${\hat{\lambda }}_{MM},$ based on the estimation ${\hat{\beta }}_{MM}$ is obtained as follows (see [23]),

(21) $${\hat{\lambda }}_{MM}=\sqrt{\frac{\Psi \left({\hat{\beta }}_{MM}+1\right)-\Psi \left(1\right)}{m_{2}}}.$$

#### 2.2.3. MLM Estimation

In this subsection, we discuss the L-moments estimators of the parameters λ and β, say ${\hat{\beta }}_{MLM}$ and ${\hat{\lambda }}_{MLM}$, respectively, when the parameter α is non-parametrically estimated by Equation (13) as ${\hat{\alpha }}_{NL}$.

The L-moments estimation method was originally introduced by Hosking [32]. The method is a more robust estimation technique than the method of moments. Some valuable properties of the L-moments estimators were shown by Hosking [32]. In order to obtain L-moments estimators of the parameters of a family of distributions with two parameters, as in the moments method, the first two sample L-moments are equated to the corresponding population L-moments and solved with respect to the parameters. However, population L-moments of the generalized Rayleigh distribution cannot be obtained analytically. By using the quadratic transformation of a generalized Rayleigh random variable, Kundu and Ragab [23] have obtained the modified L-moments estimator of the parameters β and λ. Now, let $X_{1},X_{2},\ldots ,X_{n}$ be a random sample from the ASP with parameter α and $X_{1}∼GR\left(\lambda ,\beta \right)$ and also α estimated by the estimator (13) as $\alpha_{NL}$. In this situation, we have the sample ${\hat{Y}}_{1},{\hat{Y}}_{2}\ldots ,{\hat{Y}}_{n}$ from Equation (14). By using the sample ${\hat{Y}}_{k},\left(k=1,2,\cdots ,n\right)$ and following the similar steps of the “Modified L-Moment Estimators” section given in [23], it can be written that the sample L-moments $l_{1}$ and $l_{2}$ are:

(22) $$l_{1}=\frac{1}{n}\sum_{k=1}^{n}{\hat{y}}_{\left(k\right)}^{2}$$

and:

(23) $$l_{2}=\frac{2}{n\left(n-1\right)}\sum_{k=1}^{n}\left(k-1\right){\hat{y}}_{\left(k\right)}^{4}-l_{1},$$

respectively. On the other hand, population L-moments $L_{1}$ and $L_{2}$ are:

(24) $$L_{1}=\frac{1}{\lambda }\Psi \left(\beta +1\right)-\Psi \left(1\right)$$

and:

(25) $$L_{2}=\frac{1}{\lambda }\Psi \left(2\beta +1\right)-\Psi \left(\beta +1\right),$$

respectively; see [23]. Thus, ${\hat{\beta }}_{MLM}$ can be obtained from the solution of the nonlinear equation:

(26) $$\frac{\Psi \left(2\beta +1\right)-\Psi \left(\beta +1\right)}{\Psi \left(\beta +1\right)-\Psi \left(1\right)}-\frac{l_{2}}{l_{1}}=0,$$

Therefore, using Equations (22) and (24) and estimation ${\hat{\beta }}_{MLM}$, ${\hat{\lambda }}_{MLM}$ is:

(27) $${\hat{\lambda }}_{MLM}=\frac{\Psi \left({\hat{\beta }}_{MLM}+1\right)-\Psi \left(1\right)}{l_{1}},$$

from [23].

#### 2.2.4. MLS Estimation

In this subsection, when the parameter α is nonparametrically estimated by the estimator (13), we obtain the least-squares estimators of the λ and β parameters of the ASP with the generalized Rayleigh distribution. Let $X_{1},X_{2},\cdots ,X_{n}$ be a random sample of size *n* from an ASP with the generalized Rayleigh distribution, and we indicate the estimation of the parameter α as ${\hat{\alpha }}_{NL}$. In this situation, we have the estimated observations ${\hat{Y}}_{1},{\hat{Y}}_{2}\ldots ,{\hat{Y}}_{n}$ from Equation (14). Thus, the MLS estimators of the parameters λ and β, say ${\hat{\lambda }}_{MLS}$ and ${\hat{\beta }}_{MLS}$, respectively, can be obtained by minimizing the equation:

(28) $$\sum_{j=1}^{n}{\left({\left(1-e^{-{\left(\lambda {\hat{Y}}_{\left(j\right)}\right)}^{2}}\right)}^{\beta }-\frac{j}{n+1}\right)}^{2}$$

with respect to β and λ, where ${\hat{Y}}_{\left(j\right)}$,$\left(j=1,2,\cdots ,n\right)$, indicates the $j^{\mathrm{th}}$ ordered observations of the sample ${\hat{Y}}_{1},{\hat{Y}}_{2},\cdots ,{\hat{Y}}_{n}$.

We refer the readers to [33] for further information on least-squares estimation.

The entropy measure of generalized Rayleigh distribution given by Equation (7) can be easily computed by using the (plug-in) estimators of the parameters obtained by the methods of ML, MMSP, MLS, MM, and MLM [24].

## 3. Simulation Study

**Figure 3:**
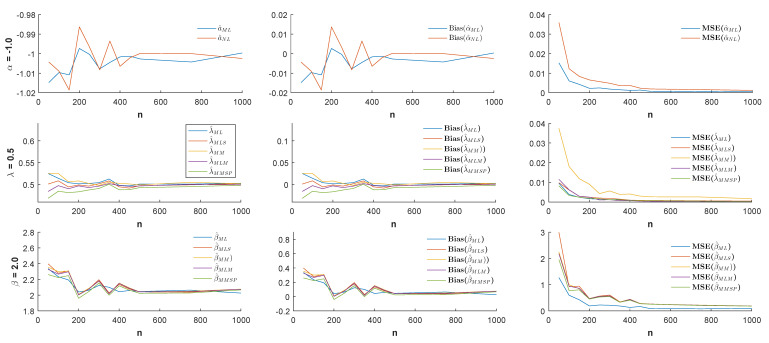
Estimates (**left**), biases (**center**), and MSE values (**right**) when the parameters α=−1.0, λ=0.5, and β=2.0.

**Figure 4:**
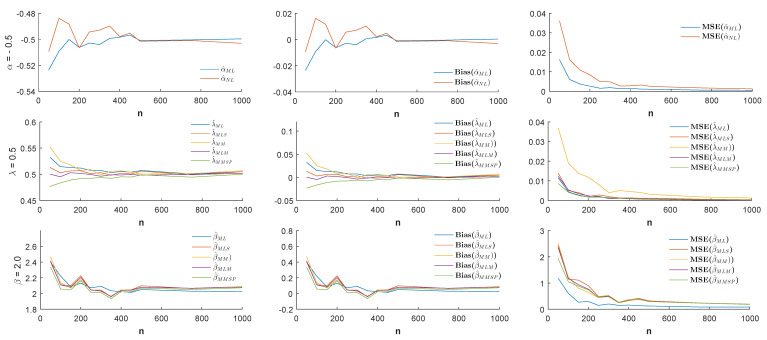
Estimates (**left**), biases (**center**), and MSE values (**right**) when the parameters α=−0.5, λ=0.5, and β=2.0.

**Figure 5:**
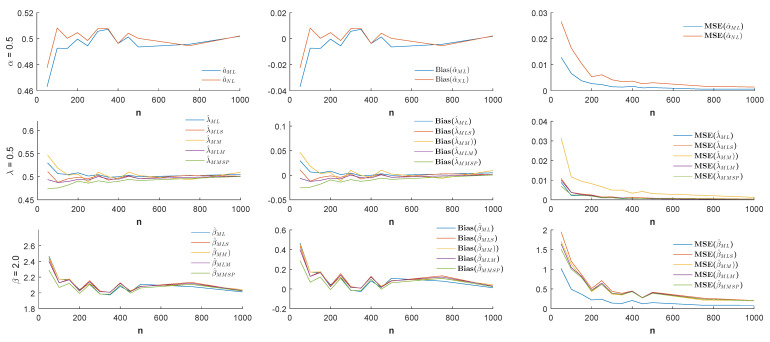
Estimates (**left**), biases (**center**), and MSE values (**right**) when the parameters α=0.5, λ=0.5, and β=2.0.

**Figure 6:**
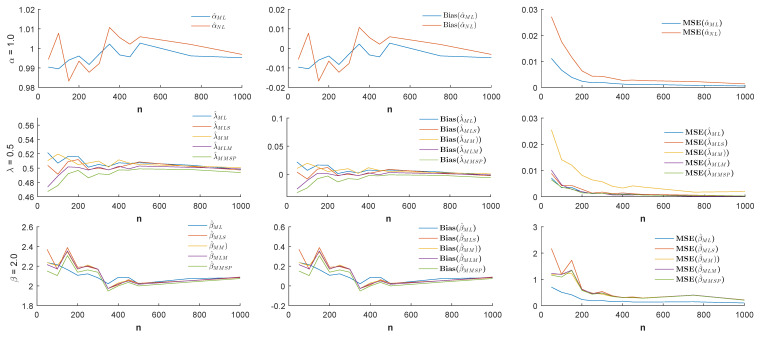
Estimates (**left**), biases (**center**), and MSE values (**right**) when the parameters α=1.0, λ=0.5, and β=2.0.

This section presents the results of some simulation studies that compare the efficiencies of the ML, MMSP, MLS, MM, and MLM estimators obtained in the previous section. In the simulation studies, the values of the parameters λ and β were set as 0.5 and 2.0, respectively, without loss of generality. For the different sample of sizes *n* $\left(n=50,100,150,\ldots ,500,750,1000\right)$ and the different values of the parameter α (α = −1.0, −0.5, 0.5, 1.0), estimates, biases, and MSE values were simulated by 1000 replicated simulations. The obtained simulation results are visualized by Figure 3, Figure 4, Figure 5 and Figure 6.

According to the visualized simulation results given by Figure 3, Figure 4, Figure 5 and Figure 6, the estimates of all estimators were quite satisfactory. In addition, the results show that when the sample size *n* increased, the biases and MSE values decreased for all estimators. Thus, it can be concluded that all estimators were asymptotically unbiased and consistent. In estimating the α parameter, the MLE estimator provided better estimation performance than the non-linear estimator ${\hat{\alpha }}_{NL}$ according to the MSE criteria. Besides, in the estimation of the parameters λ and β, the MLE and MMSP estimators outperformed the MM, the MLM, and the MLS estimators with the smallest MSE values.

## 4. Data Analysis

In this section, we present two practical applications with real-life datasets: the air-conditioning system and No. 4 datasets. In order to demonstrate the performance of ASP in modeling the successive arrival times with a monotone trend, the datasets were modeled using both the ASP with the generalized Rayleigh distribution and the RP. Before the analysis of the datasets, we investigated whether the data were consistent with a generalized Rayleigh distribution by considering the following linear regression model derived by taking the logarithm of Equation (1).

(29) $$\ln X_{k}=\tau -\alpha \ln k+\varepsilon_{k}$$

where $\tau =E\left(\ln Y_{k}\right)$ and $\varepsilon_{i},\left(i=1,2,\ldots ,n\right)$ is the error term; see [19] for further information on deriving this regression model. According to this regression model, if the exponentiated errors have the generalized Rayleigh distribution, then the data are consistent with a generalized Rayleigh distribution with parameters θ and ξ. Considering the parameter α is estimated with Equation (13), the error term $\varepsilon_{k}$ in Equation (29) can be estimated by:

(30) $${\hat{\varepsilon }}_{k}=\ln X_{k}-\hat{\tau }+{\hat{\alpha }}_{NL}\ln k,$$

where $\hat{\tau }$ is easily estimated by:

(31) $$\hat{\tau }=\frac{\ln\Gamma \left(k+1\right)\sum_{k=1}^{n}\ln X_{k}\ln k-\sum_{k=1}^{n}{\left(\ln k\right)}^{2}\sum_{k=1}^{n}\ln X_{k}}{{\left(\ln\Gamma \left(k+1\right)\right)}^{2}-n\sum_{k=1}^{n}{\left(\ln k\right)}^{2}}.$$

Therefore, the consistency of the exponentiated errors with a generalized Rayleigh distribution can be tested by using a goodness of fit test such as Kolmogorov–Smirnov (K-S). Besides, to compare the performance of ASP and RP, we used the mean-squared error (MSE*) given by:

- MSE*$=\frac{1}{n}\sum_{k=1}^{n}{\left(X_{k}-{\hat{X}}_{k}\right)}^{2},$

where ${\hat{X}}_{k}$ is calculated by:

(32) $${\hat{X}}_{k}=\left\{\begin{array}{cc} {\hat{\mu }}_{\left(ML\right)}k^{-{\hat{\alpha }}_{ML}} & \mathrm{ASP}\;\mathrm{with}\;\mathrm{the}\;\mathrm{ML}\;\mathrm{estimators},\;\;\;\\ {\hat{\mu }}_{\left(MMSP\right)}k^{-{\hat{\alpha }}_{NL}} & \mathrm{ASP}\;\mathrm{with}\;\mathrm{the}\;\mathrm{MMSP}\;\mathrm{estimators},\;\\ {\hat{\mu }}_{\left(MLS\right)}k^{-{\hat{\alpha }}_{NL}} & \mathrm{ASP}\;\mathrm{with}\;\mathrm{the}\;\mathrm{MLS}\;\mathrm{estimators},\;\\ {\hat{\mu }}_{\left(MM\right)}k^{-{\hat{\alpha }}_{NL}} & \mathrm{ASP}\;\mathrm{with}\;\mathrm{the}\;\mathrm{MM}\;\mathrm{estimators},\;\;\\ {\hat{\mu }}_{\left(MLM\right)}k^{-{\hat{\alpha }}_{NL}} & \mathrm{ASP}\;\mathrm{with}\;\mathrm{the}\;\mathrm{MLM}\;\mathrm{estimators},\\ {\hat{\mu }}_{\left(ML\right)} & \mathrm{RP}\;\mathrm{with}\;\mathrm{the}\;\mathrm{ML}\;\mathrm{estimators},\;\;\;\; \end{array}\right.$$

where the ${\hat{\mu }}_{\left(.\right)}$ notation indicates the estimate of the expected value of $X_{1}$ with the presented estimators in Section 2. Furthermore, we define the $S_{k}=X_{1}+X_{2}+\cdots +X_{k},k=1,2,\ldots ,n$. The random variable $S_{k},k=1,2,\ldots ,n$ is easily estimated by using the estimates ${\hat{X}}_{k}$ as ${\hat{S}}_{k}=\sum_{j=1}^{k}{\hat{X}}_{j}$. Thus, we can demonstrate the performances of the RP and five ASPs with the ML, MMSP, MLS, MM, and MLM estimators by plotting $S_{k}$ and ${\hat{S}}_{k}$, against k,k=1,2,⋯,n.

### 4.1. Air-Conditioning System Data

**Figure 7:**
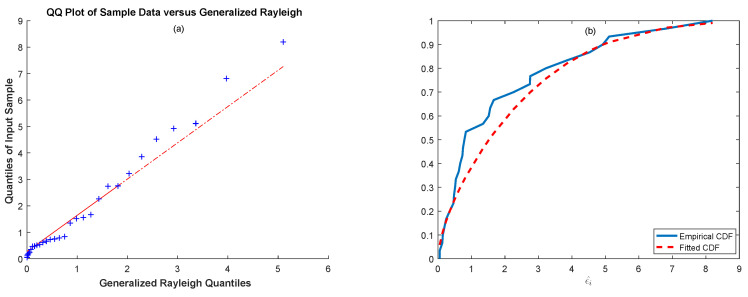
(**a**) Q-Q plot of the $\exp({\hat{\varepsilon }}_{i})$ and (**b**) empirical and fitted cdf of the $\exp({\hat{\varepsilon }}_{i})$ for the air-conditioning system data.

This dataset is related to the study of the failure times of an aircraft (Aircraft Number 7912) air-conditioning system dataset presented by Proschan [34] that includes 30 observations. For this dataset, estimations for the exponential errors were θ=0.3188 and ξ=0.2280 (K-S statistic = 0.1886, *p*-value = 0.2083). Hence, we can conclude that the data can be modeled with a generalized Rayleigh distribution. We also present Figure 7a,b, where Figure 7a illustrates the Q-Q plot of the exponentiated errors against the generalized Rayleigh distribution and Figure 7b illustrates plots of the empirical and the fitted generalized Rayleigh cdf.

**Figure 8:**
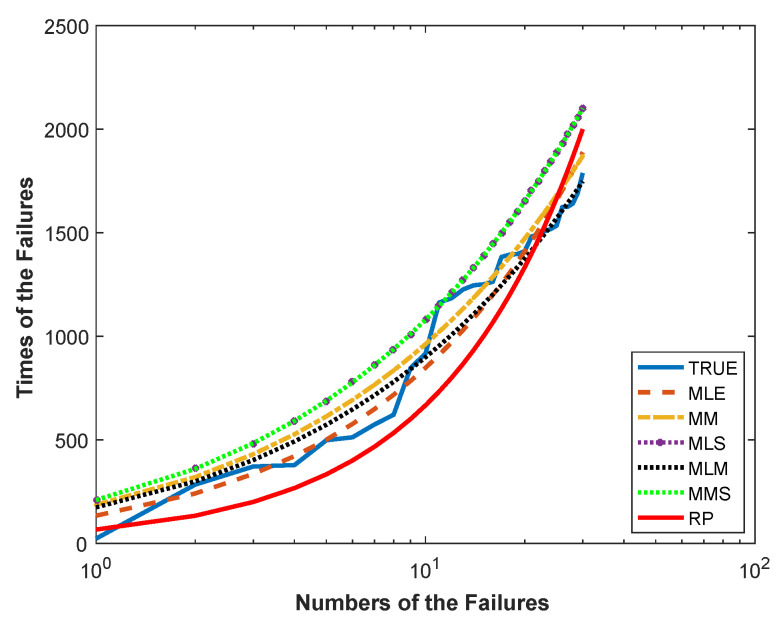
The plots of $S_{k}$ and ${\hat{S}}_{k}$ against the number of failures for the air-conditioning system data.

The MSE* values and the parameter estimates of the RP and ASPs with different estimators, the ML, the MMSP, the MLS, the MM, and the MLM, are summarized in Table 1. According to Table 1, the ASP with the ML estimators gave the best modeling performance with the least MSE* value. For this dataset, the calculated Shannon entropy with the ML estimators was also −1.2412. The relative performance of the employed ASPs and RP can clearly be seen from Figure 8, which plots $S_{k}$ and ${\hat{S}}_{k}$ versus the number of failures *k* (k=1,2,⋯,30).

As can be seen in Figure 8, the ${\hat{S}}_{k}$s estimated by the ASP with the ML estimators followed the actual failure times more closely than the other process, as consistent with the Monte Carlo simulation results presented in the previous section.

Now, let us investigate the optimal ASP considering the popular distribution models such as the generalized Rayleigh, gamma, log normal, inverse Gaussian, and Weibull distributions for modeling this dataset. For the different ASP with ML estimators, evaluated MSE* and parameter estimates are tabulated by Table 2. According to the results given by Table 2, ASP with the generalized Rayleigh distribution outperformed the other models with the least MSE* values. Therefore, it can be concluded that the ASP with the generalized Rayleigh distribution is an optimal model of the air-conditioning system data among the ASPs with the gamma, Weibull, inverse Gaussian, and log normal distribution.

### 4.2. No. 4 Data

The No. 4 dataset is related to unscheduled maintenance actions for the U.S.S. Grampus No. 4 main propulsion diesel engine [35]. The dataset contains 56 observations, which are the times between successive unscheduled maintenance actions.

**Figure 9:**
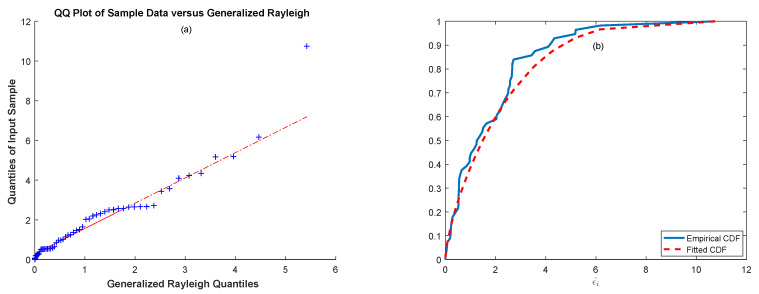
(**a**) Q-Q plot of the $\exp({\hat{\varepsilon }}_{i})$ and (**b**) empirical and fitted cdf of the $\exp({\hat{\varepsilon }}_{i})$ for the No. 4 data.

As in the previous example, we first explored whether the underlying distribution of the data was appropriate with a generalized Rayleigh distribution. For this dataset, estimations for the exponential errors were θ=0.3188, ξ=0.2280; the value of the evaluated K-S test was 0.1886; and the corresponding *p*-value was 0.2083. By considering the value of the K-S statistic and the corresponding *p*-value, we can say that the data are appropriate for the generalized Rayleigh distribution. To support this conclusion, we present Figure 9a,b, which shows the Q-Q plot of the exponentiated errors (${\hat{\varepsilon }}_{i}$) against the generalized Rayleigh distribution and the empirical and fitted cdf of the generalized Rayleigh distribution, respectively.

We can clearly see from Figure 9a that the data points fall approximately on a straight line, and the fitted cdf of the exponentiated errors closely followed the empirical cdf in Figure 9b. Thus, it can be concluded that the data can be modeled by a generalized Rayleigh distribution. For the No. 4 dataset, the ML, the MLS, the MM, the MLM, and the MMSP estimates of the parameters α, λ and β and the MSE${}^{*}$ values of the corresponding processes are tabulated in Table 3.

**Figure 10:**
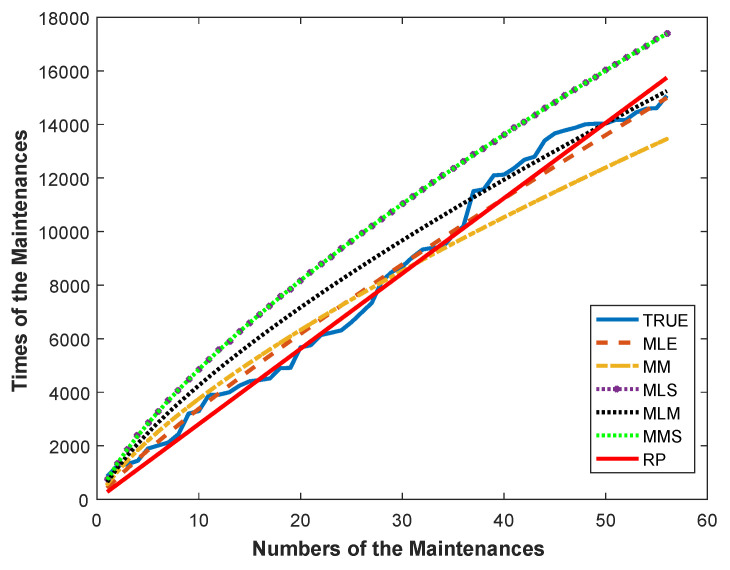
The plots of $S_{k}$ and ${\hat{S}}_{k}$ against the number of maintenance actions for the No. 4 dataset.

By Table 3, the ASP with ML estimators is an optimal process for modeling of this dataset because of it outperformed the RP and the ASPs with other estimators with a smaller MSE* value. Shannon entropy with the ML estimators was also calculated as −0.2418 for this dataset. Further, the relative performances of the mentioned ASPs and RP can be seen from Figure 10. Figure 10 plots the $S_{k}$ and ${\hat{S}}_{k}$ versus the number of unscheduled maintenance actions *k* (k=1,2,⋯,56).

As can be seen in Figure 10, the ASP with the ML estimators more fairly followed the actual values than the RP. Thus, according to Figure 10 and Table 3, it is concluded that the ASP provides a better data fit than RP. In addition, for the No. 4 dataset, the evaluated parameter estimates and the corresponding MSE* values of the alternative ASPs with the different distribution models are summarized by Table 4. By Table 4, we can say that the ASP with the generalized Rayleigh distribution is an optimal model for the No. 4 dataset, since it outperformed other ASPs with the gamma, log normal, inverse Gaussian, and Weibull distributions with a smaller MSE* value.

## 5. Conclusions

In this study, we have investigated the solution of the statistical inference problem for the ASP with the generalized Rayleigh distribution. The ASP is a useful monotonic stochastic process for successive arrival times with a trend. In the study, for the different values of the parameter α, the monotonic behavior of the ASP has been illustrated by Figure 1. In the stage of statistical inference, the estimators of the ASP parameters have been obtained by using the different estimation methods such as the ML, the MMSP, the MM, the MLM, and the MLS. To bring into the open the beneficial properties of the obtained estimators such as bias and MSE, some Monte Carlo simulation results have also been presented with different scenarios. According to the presented Monte Carlo simulation results, it can be said that all of the obtained estimators produced acceptable parameter estimates with similar accuracy from the bias and MSE point of view. In addition, by the results of the Monte Carlo simulations, it can also be concluded that all the estimators were asymptotically unbiased and consistent since their bias and MSE values decreased when the sample size increased. In terms of the convergence ratio of the estimators to the actual parameter values, it has been seen that the ML estimators converged faster to the actual values of the parameters than the modified estimators.

In the study, the real data modeling behavior of the ASP has been demonstrated with two data analyses on the air-conditioning system and No. 4 datasets. The ASP with the generalized Rayleigh distribution presented better data fits for both the air-conditioning system and the No. 4 datasets than the RP with smaller MSE* values. Besides, for both datasets, the ASP with the generalized Rayleigh distribution outperformed the alternative ASPs with the gamma, log normal, inverse Gaussian, and Weibull distributions with smaller MSE* values. Thus, it is concluded that the ASP with the generalized Rayleigh distribution provides quite satisfactory modeling performance for successive arrival times with a trend and is a powerful alternative to the ASPs with famous reliability distributions such as gamma, log normal, inverse Gaussian, and Weibull.

## Figures and Tables

**Table 1:** Parameter estimates and process comparison for the air-conditioning system dataset. MM, modified moments; MMSP, modified maximum spacing.

Process	Method	$\hat{\alpha }$		$\hat{\lambda }$		$\hat{\beta }$		MSE*/$10^{3}$		SE/$10^{2}$
ASP	ML	0.31842		0.31541		0.00354		4.5938		0.1237
	LS	0.47753		0.28424		0.00211		5.0634		0.1299
	MM			0.26388		0.00225		4.8376		0.1269
	MLM			0.20832		0.00203		4.7781		0.1262
	MMSP			0.28427		0.00211		5.0629		0.1299
RP	ML	1.00000		0.28950		0.00671		5.0451		0.1296

**Table 2:** Parameter estimates (P. Est.) and evaluated MSE* values of the different ASP models for the air-conditioning system data.

	Model									
	Generalized Rayleigh		Gamma		Log Normal		Weibull		Inverse Gaussian	
P. Est.	$\hat{\alpha }$	0.31842	$\hat{\alpha }$	0.47024	$\hat{\alpha }$	0.47736	$\hat{\alpha }$	0.42010	$\hat{\alpha }$	0.57539
	$\hat{\lambda }$	0.31541	${\hat{k}}_{G}$	0.88876	${\hat{\mu }}_{LN}$	4.54604	${\hat{\theta }}_{W}$	149.19408	${\hat{\mu }}_{IG}$	233.18956
	$\hat{\beta }$	0.00354	${\hat{\theta }}_{G}$	201.42145	${\hat{\sigma }}_{LN}$	1.25729	${\hat{\lambda }}_{W}$	0.90586	${\hat{\sigma }}_{IG}$	69.24197
MSE*/$10^{3}$	4.59384		4.79013		5.04469		4.66714		5.31385	
SE/$10^{2}$	0.12374		0.12636		0.12967		0.12472		0.13309	

**Table 3:** Parameter estimates and process comparison for the No. 4 dataset.

Process	Method	$\hat{\alpha }$		$\hat{\lambda }$		$\hat{\beta }$		MSE*/$10^{4}$		SE/$10^{2}$
ASP	ML	0.15930		0.27920		0.00102		6.6508		0.3446
	LS	0.29543		0.30790		0.00063		7.0362		0.3544
	MM			0.16680		0.00052		6.7735		0.3477
	MLM			0.25122		0.00062		6.7581		0.3473
	MMSP			0.30783		0.00063		7.0359		0.3544
RP	ML	1.00000		0.34345		0.00178		6.8774		0.3504

**Table 4:** Parameter estimates (P. Est.) and evaluated MSE* values of the different ASP models for the No. 4 dataset.

	Model									
	Generalized Rayleigh		Gamma		Log Normal		Weibull		Inverse Gaussian	
P. Est.	$\hat{\alpha }$	0.15930	$\hat{\alpha }$	0.14743	$\hat{\alpha }$	0.29649	$\hat{\alpha }$	0.15118	$\hat{\alpha }$	0.97090
	$\hat{\lambda }$	0.27920	${\hat{k}}_{G}$	0.94236	${\hat{\mu }}_{LN}$	5.88128	${\hat{\theta }}_{W}$	420.69967	${\hat{\mu }}_{IG}$	5904.50655
	$\hat{\beta }$	0.00102	${\hat{\theta }}_{G}$	445.14408	${\hat{\sigma }}_{LN}$	1.38547	${\hat{\lambda }}_{W}$	0.97900	${\hat{\sigma }}_{IG}$	667.82473
MSE*/$10^{4}$	6.65086		6.65534		8.67224		6.65311		79.32288	
SE/$10^{2}$	0.34462		0.34474		0.39352		0.34468		1.19016	
